# Viruses: A Natural History

**DOI:** 10.3201/eid2911.231225

**Published:** 2023-11

**Authors:** Stephen S. Morse

**Affiliations:** Columbia University Mailman School of Public Health, New York, New York, USA

**Keywords:** viruses, influenza, COVID-19, respiratory infections, severe acute respiratory syndrome coronavirus 2, SARS-CoV-2, SARS, coronavirus, coronavirus disease, vector-borne infections, zoonoses, Roossinck, book review

Because of the COVID-19 pandemic that claimed ≈7 million lives worldwide, it is easy to lose sight of the great variety and many roles of viruses in the world and just view them as sinister agents of disease. Fortunately, Dr. Marilyn Roossinck, professor emerita of virus ecology at Pennsylvania State University, is here to restore our perspective and redress the balance in Viruses: A Natural History (Figure). Clearly, viral diseases often have the most dramatic effects and receive the greatest attention, and Dr. Roossinck does not neglect them. However, she elegantly demonstrates that viruses are far more than causes of disease. Viruses are enormously varied and indispensable to our global ecology; they drive many essential biological processes, including maintaining the carbon cycle in the oceans. She notes that many, perhaps most, viruses do not cause observable disease in their hosts and addresses the often asked question of whether viruses can be beneficial, providing examples of benefits. Many other aspects of the virus universe are explored by using clear prose and stunning graphics.

The stunning graphic design is one of the outstanding features of the book, and, although it might cause distraction, I think it is a powerful way to engage the reader. The illustrated vignettes are also a way to begin for persons who prefer to wade into the material more gradually before plunging into deeper technical details. The graphic design is carried throughout the 9 sections of the book: Introduction, The Depth and Breadth of Viruses, Viruses Making More Viruses, How Viruses Get Around, Evolution, The Battle Between Viruses and Hosts, Viruses in Ecosystem Balance, The Good Viruses, and The Pathogens. Dr. Roossinck considers whether viruses have colors, setting the stage for the visually impressive graphics to come, many of which are false-color representations of virus structures. Whether viruses have color is also a preview of the thought-provoking points she brings up along the way. Given Dr. Roossinck’s background as a plant virologist, the plant kingdom receives its share of attention, as do viruses of bacteria, archaea, and fungi.

This is not your conventional virology textbook, although the reader does get a solid foundation in virology. I would recommend it especially for students, young adults, and curious general readers, but nuggets of information are there for even the seasoned virologist. For example, RNA viruses, so common and concerning in eukaryotes, oddly seem exceedingly rare in prokaryotes, notwithstanding greater diversity and evolutionary history. A virus might even have been responsible for our own existence; placental mammals might have evolved through integration of an endogenous retrovirus gene (actually a gene family). Speak of a virus changing the world!

The integration of arresting images and text, especially technical detail, is always a challenge and works better in some places than others. The book ends rather abruptly with a vignette of African swine fever virus and might have benefited from a concluding section. However, those are small quibbles for a book that offers us a rare portal into the complexity and subtlety of the world of viruses.

**Figure:**
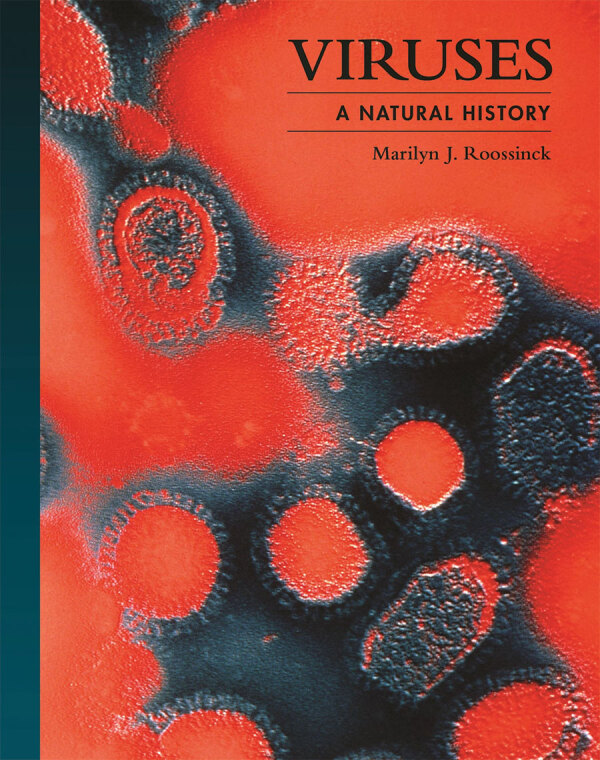
Viruses: A Natural History

